# The Proteins API: accessing key integrated protein and genome information

**DOI:** 10.1093/nar/gkx237

**Published:** 2017-04-05

**Authors:** Andrew Nightingale, Ricardo Antunes, Emanuele Alpi, Borisas Bursteinas, Leonardo Gonzales, Wudong Liu, Jie Luo, Guoying Qi, Edd Turner, Maria Martin

**Affiliations:** EMBL-EBI, Wellcome Genome Campus, Hinxton, Cambridgeshire CB10 1SD, UK

## Abstract

The Proteins API provides searching and programmatic access to protein and associated genomics data such as curated protein sequence positional annotations from UniProtKB, as well as mapped variation and proteomics data from large scale data sources (LSS). Using the coordinates service, researchers are able to retrieve the genomic sequence coordinates for proteins in UniProtKB. This, the LSS genomics and proteomics data for UniProt proteins is programmatically only available through this service. A Swagger UI has been implemented to provide documentation, an interface for users, with little or no programming experience, to ‘talk’ to the services to quickly and easily formulate queries with the services and obtain dynamically generated source code for popular programming languages, such as Java, Perl, Python and Ruby. Search results are returned as standard JSON, XML or GFF data objects. The Proteins API is a scalable, reliable, fast, easy to use RESTful services that provides a broad protein information resource for users to ask questions based upon their field of expertise and allowing them to gain an integrated overview of protein annotations available to aid their knowledge gain on proteins in biological processes. The Proteins API is available at (http://www.ebi.ac.uk/proteins/api/doc).

## INTRODUCTION

Discovering and understanding biological processes and diseases can be enormously cumbersome, requiring the integration and analysis of a large number of observations from world-wide produced experimental data and information collected and curated in biological resources; having access to resources that collate and interpret biological data as meaningful meta information is now essential for the scientific discovery process.

The Universal Protein Resource (UniProt) (1) is a comprehensive resource for protein sequence and annotation data. It is composed of manually curated and reviewed UniProtKB/Swiss-Prot and unreviewed, automatically annotated, UniProtKB/TrEMBL protein sequences. The UniProt Knowledgebase (UniProtKB) annotations provides detailed sequence positional functional information of protein entries along with cross-references to over 150 databases acting as a central hub of protein information. These positional features include, for example, active site residues, binding sites, disulfide bonds, regions and domains. UniProt collaborates with other bioinformatics resources, such as genomics resources Ensembl (2) and ClinVar (3) and proteomics resources PRIDE (4), PeptideAtlas (5,6) and MaxQB (7) to provide mappings between the resources and the large scale experimental data sets they provide. Data integration enables users to conduct analysis using far broader data to gain a greater overview of the biological processes they are investigating. Not all this information can be accessed programmatically. Therefore, new methods of access to this data, additional data from new resources and greater flexibility to search for specific data points are required. Web service Application Programming Interfaces (APIs) can resolve the current short comings. Representational State Transfer (REST) (8) is a framework for programmatic access to web services. Its emphasis on a dependency independent interface that is simple for non-experts to quickly take advantage of. Results are returned in consistent formats such as JSON and XML that further simplify the subsequent steps of analysis. REST web service APIs remove the burden for researchers to be expert programmers and allow them to concentrate on the scientific discovery process making it an ideal solution for providing programmatic access to biological data.

Thus, we have developed the Proteins API, a REST web service, to provide programmatic access to protein sequence information and additional resources such as genomic coordinates mapping, antibody antigen sequences and mapped proteomics sequencing peptides; to enable researchers to visualize and integrate a broader range of biological data in to their analyses. The Proteins API has already been utilized to provide positional annotations to the UniProt feature viewer, via the BioJS (9) interactive graphical representation of protein annotations ProtVista (10).

## ARCHITECTURE, DESIGN AND IMPLEMENTATION

The Proteins API has been designed to utilise the best faceted search technologies available but with a level of abstraction so that the service is easily extendable to take advantage of new technologies and add services, and are adaptable and flexible to user requests. The service has three abstract layers: (i) document storage and retrieval, (ii) indexing and search engines and (iii) client interfaces. (a) Services such as, *Proteins, Features* and *Coordinates* document storage and retrieval is implemented using the Apache Avro data serialisation framework (https://avro.apache.org) through the distribute key-value storage system Voldemort (http://www.project-voldemort.com). (b) Indexing and searching has been implemented using optimised Apache Solr (http://lucene.apache.org/solr) and *Taxonomy* utilises the Neo4J graph database (https://neo4j.com) for both its document store and indexing due to the hierarchical nature of the data; with the declarative graph query language Cypher used for searching and updating of the taxonomy graph. By example, the Cypher search queries can retrieve hierarchical information (children, parent, siblings), taxonomical lineage or relationships or a common ancestor between any two organisms. (c) Client interfaces are provided through RESTful web services using the Jersey framework (https://jersey.java.net) within a Grizzly Java framework HTTP server (https://grizzly.java.net) as the Grizzly framework resolves issues with long running HTTP transactions via response suspend/resume facilities and support for inbound and outbound non-blocking IO streams. A web user interface (UI) for conducting test queries is provide through the Swagger™ (http://swagger.io) API.

## DATA COVERAGE

The Proteins REST services cover key protein and genomic data. Positional and functional protein annotations are derived from manually reviewed and computationally inferred sequence ‘features’ from UniProt/Swiss-Prot entries, and their isoforms, and from UniProt/TrEMBL entries, respectively. Services such as *Proteomes* and *Taxonomy* data sets are based upon the relevant UniProtKB databases; whilst, genomic coordinates are calculated from the latest Genome Reference Consortium (GRC) (11) assembles available through Ensembl (2). The *Variation* service data is based on UniProtKB natural variants and mapped from large scale data sources (LSS) such as 1000 Genomes (12), ExAC (13) and COSMIC (14) and *Proteomics* data is mapped from PeptideAtlas (5,6), MaxQB (7) and EPD (15), for a proteomics service. *Antigen* service is based upon the antigen to antibody mapping from the Human Protein Atlas (HPA) (16). Data derived from sources external to the UniProtKB is only available programmatically through the service.

## SERVICES DESCRIPTION

**Figure 1.**
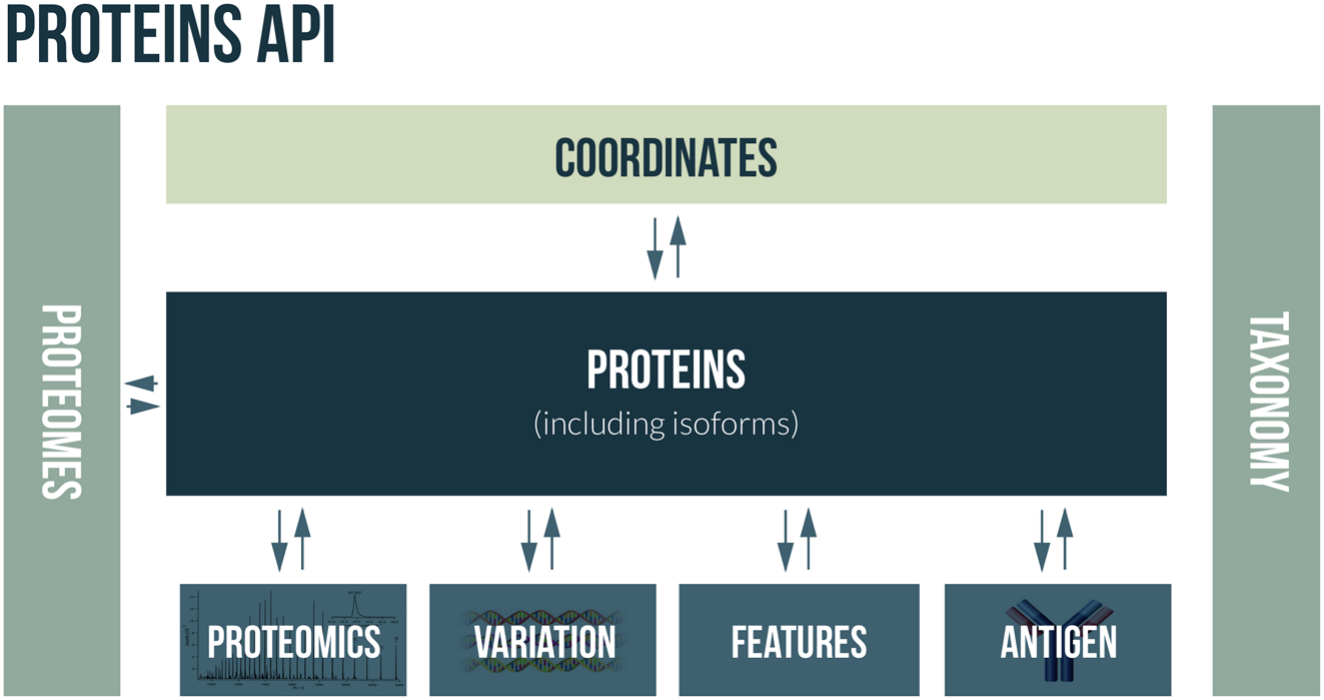
The Proteins API services and their data exchange relationship. Starting from any service a user can retrieve information from another service using inter-relationships between the services. To illustrate, Proteomes defines the set of expressed Proteins in an organism. Proteins are composed of peptide sequences available from Proteomics. Proteins have functional sequence positional annotations called Features. Proteins sequences can contain variants that can be found using the Variation service. Antigen defines regions of a protein that antibodies have been produced against. Taxonomy is a service that can be used to 1. find the relationship between different organisms and 2. The taxonomical identifier for an organism that can be used to retrieve data from all the other services except Antigen as the Antigen service currently only covers human.

An overview of the services is available via the Proteins API website. Each service can be treated as an independent entity which provides unique information specific to that service. However, each service is also related to the other services both at the biological level and with the type of data and identifiers provided in each service; this schematic coupling is demonstrated in Figure 1. *Proteins* can be used to obtain a set of UniProt entries by UniProt accessions or searching for all entries with a specific database cross reference. Equivalent UniProt entries can be generated for an accession's isoform sequence(s). *Proteomes* provides access to UniProtKB proteomes. You can search for reference using, for example, the UniProt proteome identifier, species names or taxonomy identifiers. The *Features* service provides access to UniProtKB reviewed functional annotations ‘features’ for UniProt/Swiss-Prot canonical and isoform sequences, when available, and automatically annotated for UniProt/TrEMBL entries features such as domains, regions, binding sites, active sites and glycosylation sites. *Proteomics* can be used to retrieve LSS mass spectrometry protein sequencing derived peptides for an UniProtKB sequences including isoforms. *Antigen* maps known antigen sequences from the Human Protein Atlas to UniProtKB reference proteome sequences. *Variation* is a utility to retrieve UniProtKB and LSS protein altering variants, with options to search by disease name, OMIM identifier or organism taxonomy identifier. *Coordinates* is a utility for retrieving the mapping of UniProtKB sequences and annotations to their genomic coordinates provided by Ensembl. *Taxonomy* provides access to the UniProt taxonomy database consisting of the taxonomic classification of organisms in a hierarchical tree structure. The service can be queried with taxonomy identifier(s) to retrieve hierarchical information (children, parent, sibling nodes), taxonomic lineage, relationships between nodes, common ancestors for nodes and all taxonomy nodes in the paths above or below the query. The services can also be queried with a taxonomy name to find all related information to that node or hierarchical information about its children, parent and sibling nodes.

## DATA SEARCH AND RETRIEVAL

Specific faceted searches have been implemented in each of the eight RESTful services, Table 1. Querying options and applicable filters are dependent upon the data available from each service. Generic query options such as searching by a single accession or by a list of accessions, taxonomy identifier or organism name are available in multiple services. The UniProt proteome identifier (UPID) query option is specific to the *Proteomes* and *Proteomics* services as this identifier is unique to the data sets available through these services. Predefined filters have been added to each service endpoint that are specific to the data serve by that service, such as, with the *Variation* service which has multiple filters for tailoring the set of variants returned, for example, returning variants from specific variant databases or limiting to specific HGVS variant consequence type. Where applicable the searches can be used to retrieve data for reviewed UniProt/Swiss-Prot canonical and isoform and UniProt/TrEMBL sequences depending upon the search arguments and filters applied. Due to the potential for large result sets from the *Variation, Proteomics* and *Proteomes* services, an option to paginate the results is available to users to get improved responses from the services and manage their resources for any processing of search results. Search results can be retrieved in general in JSON, XML or GFF format with the additional option of returning sequence results sets from the proteins service in FASTA format.

**Table 1.** Currently supported Proteins API Get endpoints

API end points	URLs (after: http://www.ebi.ac.uk/proteins/api)	Description summary
Proteins (including isoforms)	/proteins	Get list of UniProt entries
	/accession	Get UniProt entry by accession
	/accession/isoform	Get UniProt isoform entries from parent normal entry accession
	/db/{dbtype}:{dbid}	Get UniProt entries by UniProt cross reference and its id
	/proteomes	Search Proteomes
	/proteomes/genecentric/{upid}	Fetch proteome gene centric proteins by proteome upid
	/proteomes/proteins/{upid}	Fetch proteome proteins by proteome upid
	/proteomes/{upid}	Fetch proteome by proteome upid
	/features	Get features of list of UniProt entries
	/features/type/{type}	Search for features of given type and search terms.
	/features/{accession}	Get UniProt features by accession
	/proteomics	Get proteomics peptides of list of UniProt accessions.
	/proteomics/{accession}	Get proteomics peptides mapped to UniProt by accession
	/variation	Get variation by search
	/variation/{accession}	Get UniProt variation features by accession
	/antigen	Get antigen of list of UniProt accessions.
	/antigen/{accession}	Get proteomics peptides mapped to UniProt by accession
Coordinates	/coordinates	Query for entries with genomic location
	/coordinates/{accession}	Get genome coordinates by accession
	/coordinates/{taxonomy}/location/{gstart}-{gend}	Get genome coordinate by location
Taxonomy	/taxonomy (followed by:)	
	/ancestor/{ids}	Returns the lowest common ancestor (LCA) of two taxa
	/id/{id}	Returns details about a taxonomy, its parent, siblings and children taxa
	/id/{id}/children	Returns a list of children taxa that belongs to a taxonomy
	/id/{id}/node	Returns details about a taxonomy
	/id/{id}/parent	Returns details about the parent
	/id/{id}/siblings	Returns a list of sibling taxa
	/ids/{ids}	Returns details on taxa parents, siblings and children taxa
	/ids/{ids}/node	Returns taxa details such as the rank, mnemonic, scientific name and common name
	/lineage/{id}	Returns the taxonomic lineage
	/name/{name}	Returns a list of taxonomy with the specific queried name
	/name/{name}/node	Returns a list of taxa with a specific name
	/path	Returns all taxonomic nodes that have a relationship with the queried taxonomy ID in a specific direction (TOP or BOTTOM) and depth level
	/relationship	Returns the path between two taxa

## API DOCUMENTATION AND WEB INTERFACE

**Figure 2.**
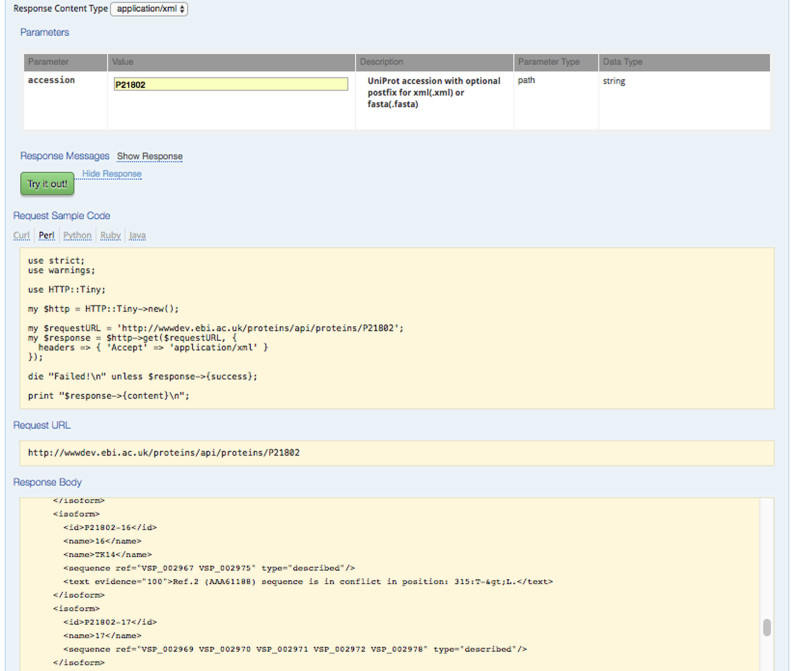
Proteins API documentation for the proteins accession search end point. The Swagger™ API generates an interactive webpage where users can ‘try out’ the service with real queries. Results are returned in the ‘Response Body’ in the user selected response format (XML illustrated). The Proteins API webpages provide users with dynamically generated source code for common scripting and programming languages with their query options and filters fully integrated into the source code that can be used as standalone scripts or programs or integrated into users’ large projects (Perl source code illustrated).

Proteins API documentation is generated using the Swagger™(http://swagger.io) API framework (http://www.ebi.ac.uk/proteins/api). Services are collated into four service groups: *Proteins, Proteomes, Coordinates* and *Taxonomy* based upon the biological data available from the services, Figure 1. We use Swagger to define a standard language agnostic interface to the RESTful APIs allowing users to discover and utilize the service without access to the source code. Documentation generated by Swagger lists all the available end-points, definitions and descriptions of the methods within each end point, and required and optional parameters and filters, Table 1. The webpages also provide an interactive interface, allowing for the execution of services thereby allowing users with little or no programming experience, to use the web services, to quickly and easily become familiar with the Proteins API services, learn how to formulate queries and execute searches. Results from executed searches are displayed within the same page in the available output formats. More advanced users interested in executing their queries in a certain programming or scripting language can get Swagger dynamically generated source code for Perl, Python, Ruby and Java, Figure 2. Queries options and filters applied in the Proteins API documentation webpages can be found in the Proteins API home page.

## EXAMPLE USE CASE

The REST services provide a flexible interface into multiple aspects of protein science; for example, details on the genetic origins of Wilson disease (OMIM:277900) (17), which proteins are implicated in the disease, how the genetic variants potentially alter the normal function of the proteins and how this compare to the mouse equivalent proteins and genes. The *Variation* service can be used to retrieve all entries with Wilson disease associated variants from the set of human genetic variants; which results in a single entry human Copper-transporting ATPase 2, ATP7B gene, (P35670). Wilson disease variants are distributed throughout the protein with no obvious variation ‘hotspot’ for the disease. Sequence annotations for P35670 can be retrieved using the *Features* service and analyzed to determine if any of the variants align to functionally important protein features such as binding, or active site. This copper transporting protein contains six copper binding sites and one active site, there is a mixture of UniProt reviewed and large scale variants found at the copper binding site residues, including a COSMIC (COSM307382) large scale study missense variant at the active site that alters the wild type aspartic acid at residue 1027 to a histidine (p.D1027H). Unique *Proteomics* peptides are found within the protein that can be used to identify P35670 in mass-spectrometry sequencing, with seven unique peptides upstream of the active site (supplementary data).

The REST service is not dedicated to human annotation alone. It is possible to retrieve the same data types: variants, features and peptides for any species in UniProtKB. Therefore, comparative analysis can be conducted between protein orthologs. Using the *Proteins* service you can search by gene name to find all ATP7B protein entries. In this case, 140 proteins are returned by the service (supplementary material); the results are paginated in this case so not all the proteins are available in the Swagger UI response body unless pagination is turned off. The human ATP7B gene (P35670) has a mouse orthologue Q64446 that share 83.59% sequence identity when analysed using separate alignment tools like Clustal Omega (supplementary data).

The *Coordinates* service can then be used to retrieve genome coordinates for both proteins so we can determine how similar the orthologue genes are to each other by looking at the exon structure of the genes transcripts and if protein sequence features exist in the same exon (supplementary data). Both P35670 and Q64446 genes consist of 21 exons with the active site residue in both proteins being found in the fourteenth exon. Therefore, we can be confident that other sequence features are related between the two proteins.

By using the sequence alignment, variants for both proteins retrieved using the *Variation* service and peptides for both proteins from the *Proteomics* service we can further examine the two proteins for similarities or differences to determine if mouse could have an orthologue phenotype to Wilson disease. The mouse protein (Q64446) has significantly less variants with only a single UniProt reviewed variant however there are a number of large scale variants that can be mapped to equivalent variants in the human protein. For example p.R1040K (rs239532167) is equivalent to the reviewed and large scale study variant p.R1038K (rs59959336) which is associated to Wilson disease and is close proximity to the active site residue in both proteins. Moreover, both proteins have a matching unique proteomics peptide between residues 1042–1054 and residues 1044–1057 in P35670 and Q64446 respectively (supplementary data). Thus, here we have been able to show how the data from the individual REST services can be combined to data mine and gain knowledge about proteins across species, in this case showing that mouse has a Wilson disease orthologue phenotype and an equivalent missense variant to look for.

## DISCUSSION

Primarily the Proteins API goal was to provide free reliable, flexible and extendable programmatic access to the rich and diverse annotations available in UniProtKB whilst making new biological data types such as genomic coordinate and proteomics peptide mapping data programmatically available. With the use of appropriate big data indexing, searching and storage technologies the Proteins API has achieved mean response times of 40 milliseconds and a peak of 200 requests processed per second when tested with over forty thousand consecutive requests. Consecutively, the Proteins API had to facilitate easy integration of its services into third party pipelines and web resources for further interpretations of the data in parallel with users’ own data. By providing a web interface to the services users can ‘talk’ to the services and thereby quickly and easily become familiar with the Proteins API services, learn how to formulate queries and with the provision of dynamically generated programming and scripting languages source code integrate the services into their resources for automatic data retrieval. This is illustrated with the BioJS (9) web component ProtVista (10) that provides an integrated overview of the data available through the Proteins API and has been added to the UniProt website as the Feature Viewer. Thus, the Proteins API services provides programmers, data scientists and researchers a new resource to search, integrate, analyze and interpret protein biological data previously unavailable to the scientific community improving the rate that experimental data can be analyzed and increasing the annotations available to understand the biological question being asked.

## AVAILABILITY

The Proteins API is available at http://www.ebi.ac.uk/proteins/api/doc.

## Supplementary Material

Supplementary Data
