# Supplementary material for: The Proteins API: accessing key integrated protein and genome information
Source: Nucleic Acids Res. 2017 Apr 5;45(Web Server issue):W539–44. doi: 10.1093/nar/gkx237 (PMC5570157; doi:10.1093/nar/gkx237)
Supplement: Supplementary Data [file gkx237_Supp.pdf]

Supplementary Data for Wilson Disease Example Use Case

Step 1: Search for proteins associated with Wilson disease

Here the *Variation* service was used to find any protein that is associated with Wilson disease and any associated variants. Figure 1 (a and b) shows the Swagger User Interface (UI) for the *Variation* service with Wilson disease added to the disease search field. Figure 1b illustrates the results after execution of the search in the response body, with a Curl command defined that is also available in Figure 2 with Figure 3 defining a Python script to execute this search.

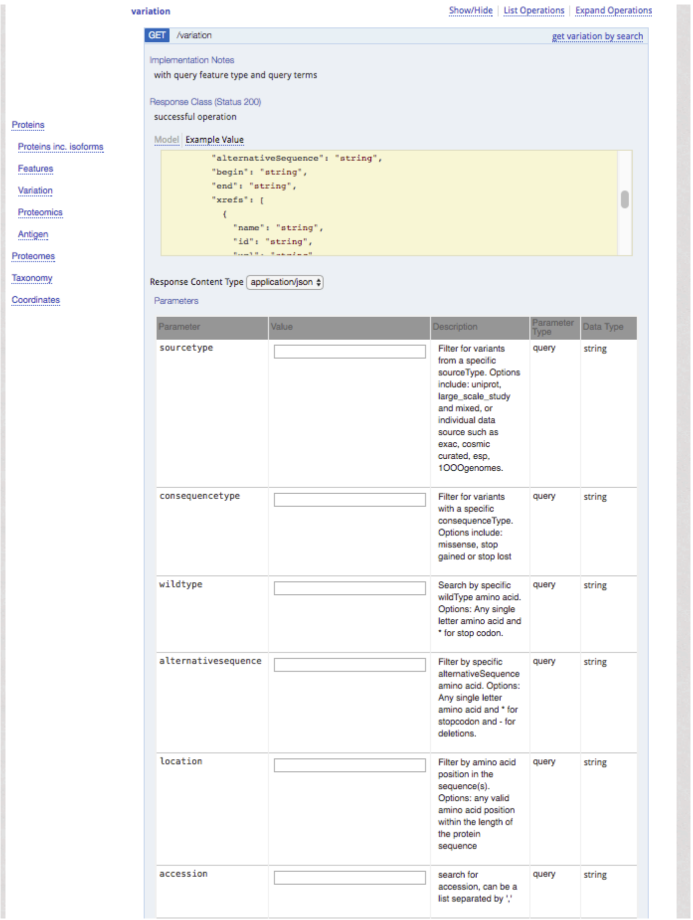

Figure 1a: Variation service Swagger UI top.

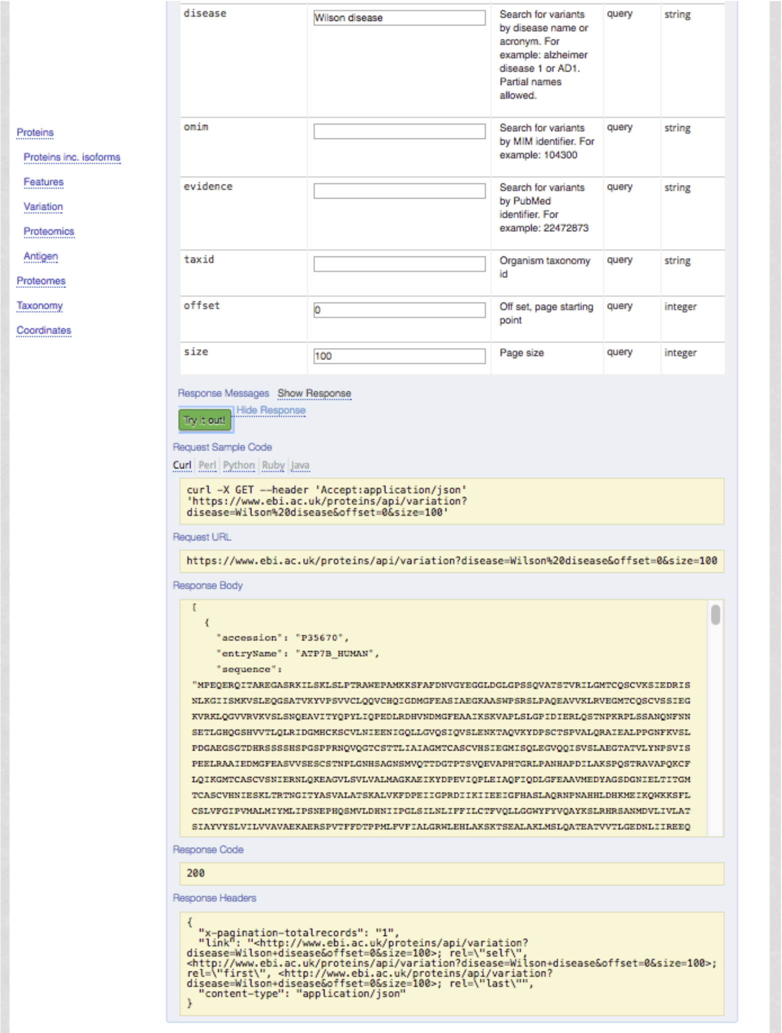

Figure 1b: *Variation* service Swagger UI bottom with Wilson disease added to the disease search field. The search has already been executed by clicking on ‘Try Out’ and the results of the search are available in JSON in the ‘Response Body’.

```
curl -X GET --header 'Accept:application/json'
'https://www.ebi.ac.uk/proteins/api/variation?disease=Wilson%20di
sease&offset=0&size=100'
```

Figure 2: Curl command to execute the search for proteins and variants annotated with being associated to Wilson disease. Pagination has been set but as can be seen in Figure 1b this search only returns a single page.

```
import requests, sys

requestURL =
"https://www.ebi.ac.uk/proteins/api/variation?disease=Wilson%20di
sease&offset=0&size=100"

r = requests.get(requestURL, headers={ "Accept" :
"application/json"})

if not r.ok:
  r.raise_for_status()
  sys.exit()

responseBody = r.text
print(responseBody)
```

Figure 3: Python script equivalent to the curl command in Figure 2 for executing the search for proteins and variants annotated with being associated to Wilson disease. Pagination has been set but as can be seen in Figure 1b this search only returns a single page.

Step 2: Searching for positional annotations 'Features' in P35670 that could be affected by Wilson disease associated variants.

The *Features* service was used to find any positional annotations in the region of the Wilson disease associated variant. Figure 4 shows the Swagger UI for the *Features* service with protein accession P35670 added to the accession search field. Figure 5 illustrates the results after execution of the search in the response body, with a Curl command defined that is also available in Figure 6 with Figure 7 defining a Python script to execute this search. An active site feature is annotated at aspartic acid 1027 is coincides with a Wilson disease associated variant p.Asp1027His (COSM307382), Figure 8.

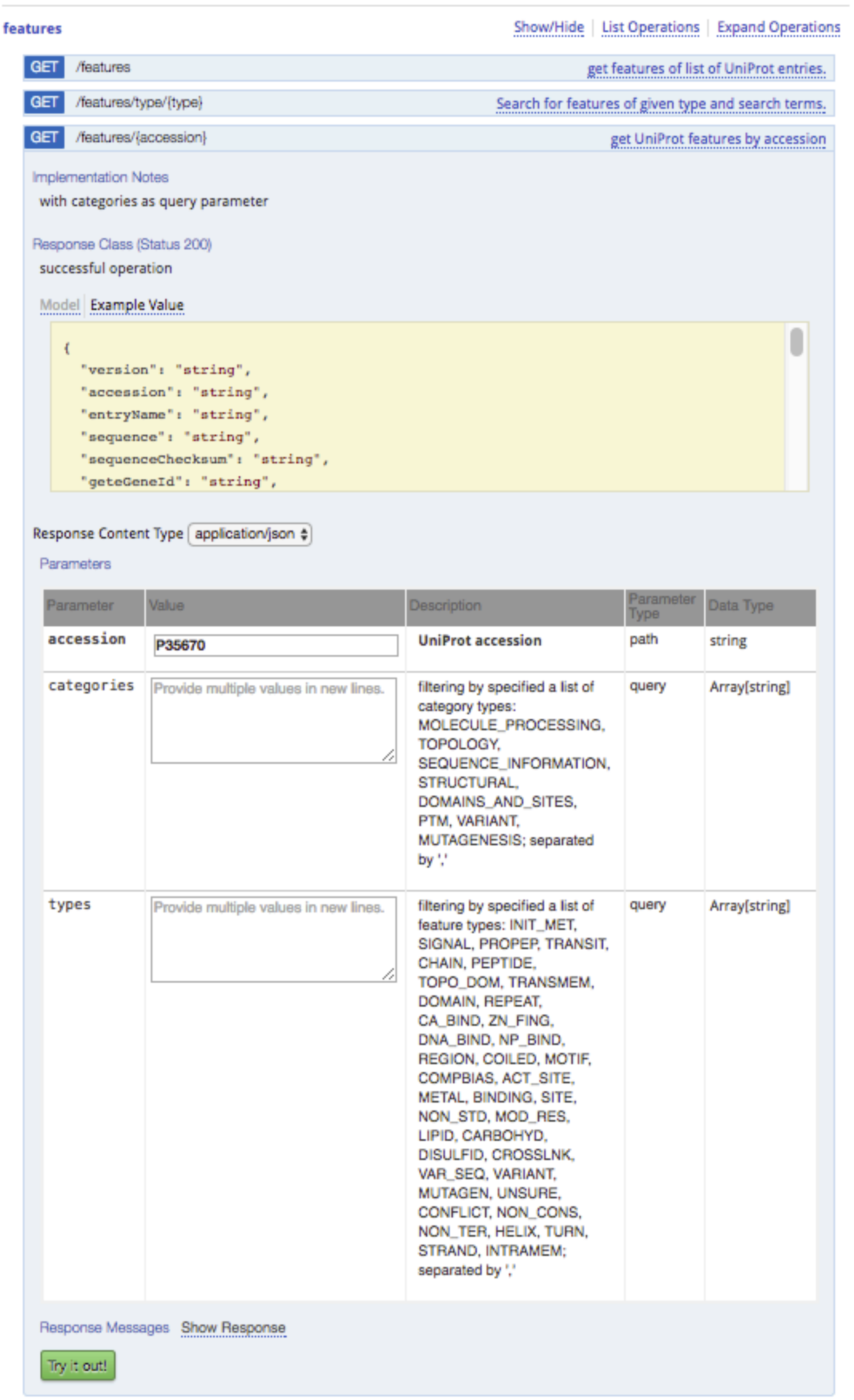

Figure 4: *Features* service search by accession. Here we are searching for all positional annotations available for the protein P35670 but it is possible to use the optional search fields to restrict to specific types of ‘features’.

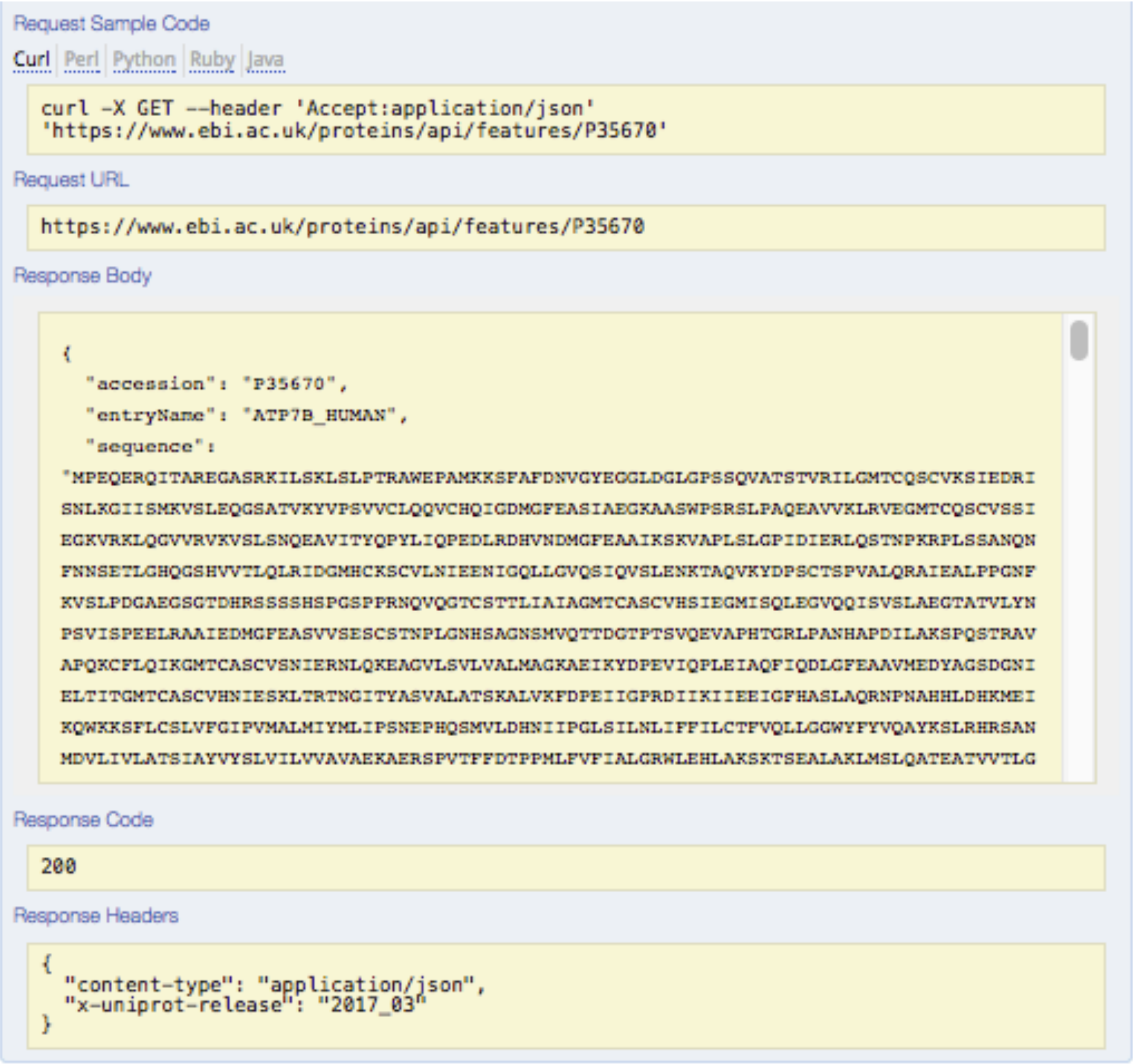

Figure 5: Results from the search for ‘features’ in P35670.

```
curl -X GET --header 'Accept:application/json'
'https://www.ebi.ac.uk/proteins/api/features/P35670'
```

Figure 6: Curl command to execute the search for protein features dynamically generated by the Swagger UI as illustrated in Figure 5.

```
import requests, sys

requestURL =
"https://www.ebi.ac.uk/proteins/api/features/P35670"

r = requests.get(requestURL, headers={ "Accept" :
"application/json"})

if not r.ok:
  r.raise_for_status()
  sys.exit()

responseBody = r.text
print(responseBody)
```

Figure 7: Python script equivalent to the curl command in Figure 6 for executing the search for proteins features in P35670.

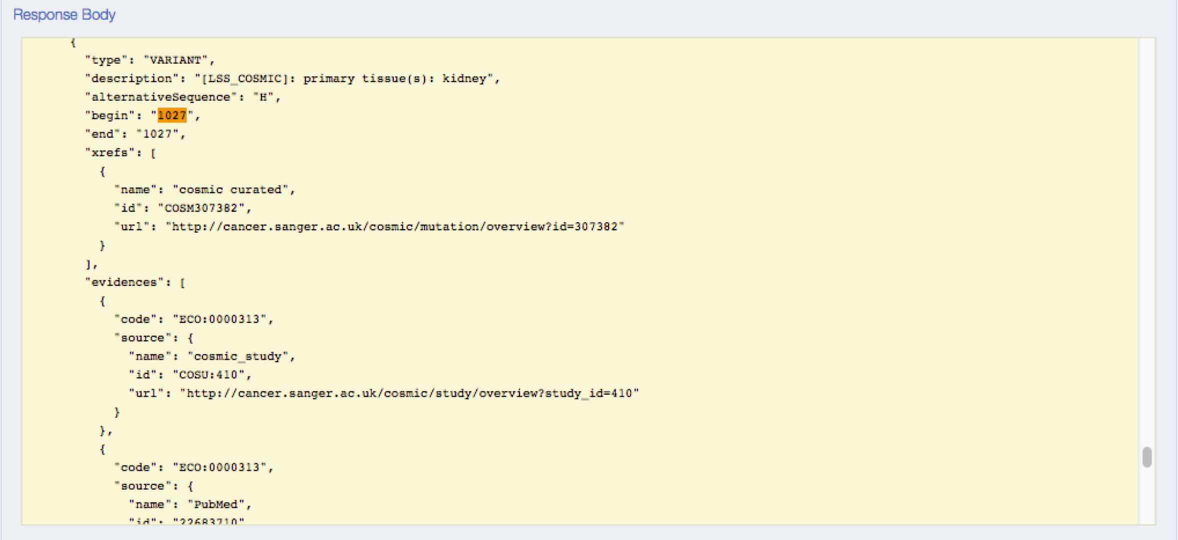

Figure 8: Identification of a variant at residue 1027 that is associated to Wilson disease and a COSMIC cancer associated variant from the large scale study variant data sets. Aspartic acid 1027 has been annotated as the active site residue.

Step 3: Proteomics Search for unique peptides around residue aspartic acid 1027 in P35670.

The *Proteomics* service was used to find any unique peptides in the region of residue Aspartic acid 1027. Figure 9 shows the Swagger UI for the *Proteomics* service with protein accession P35670 added to the accession search field. Figure 10 illustrates the results after execution of the search in the response body, with a Curl command defined that is also available in Figure 11 with Figure 12 defining a Python script to execute this search. Table 1 summarises the seven unique peptides found upstream of the active site at aspartic acid 1027.

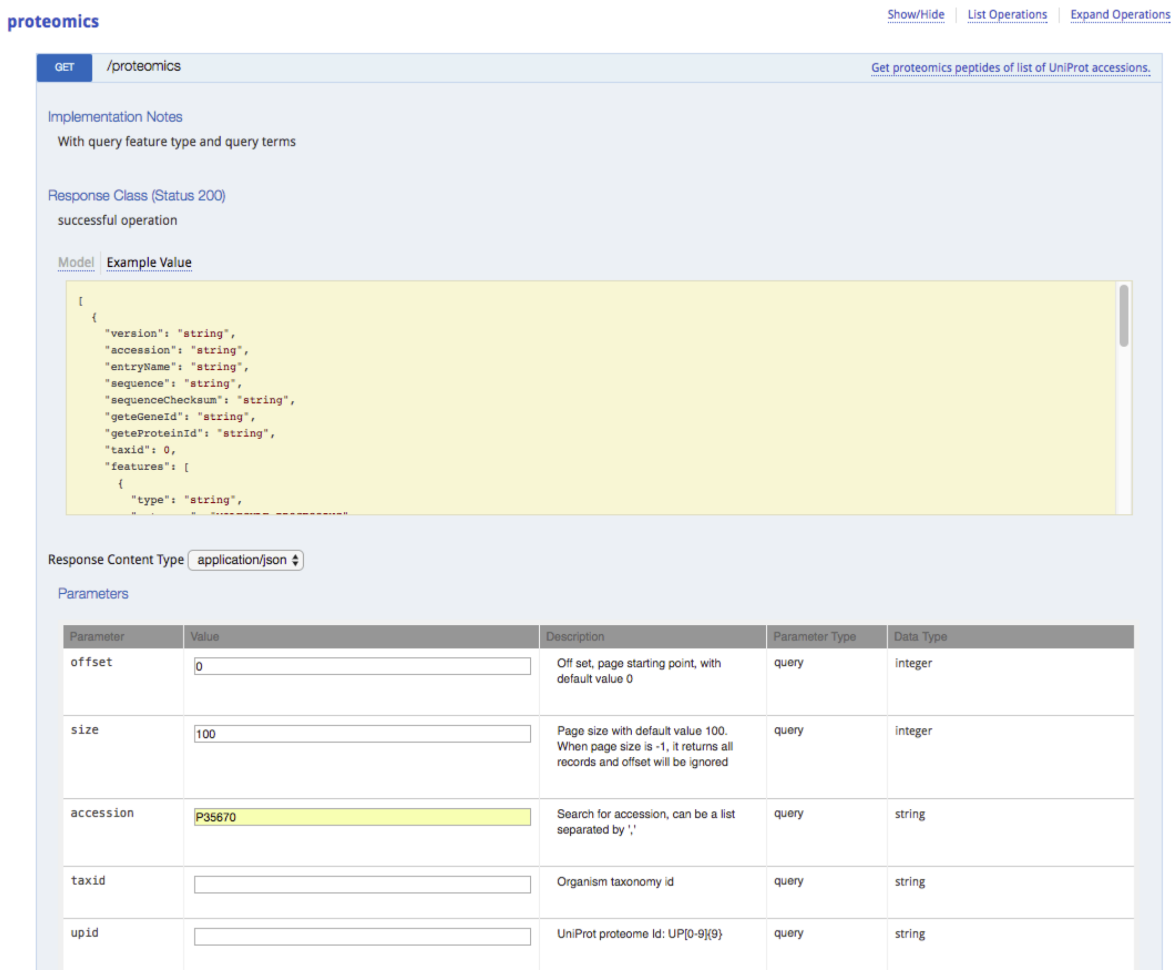

Figure 9: *Proteomics* service Swagger UI with 'P35670' entered in to the accession search field.

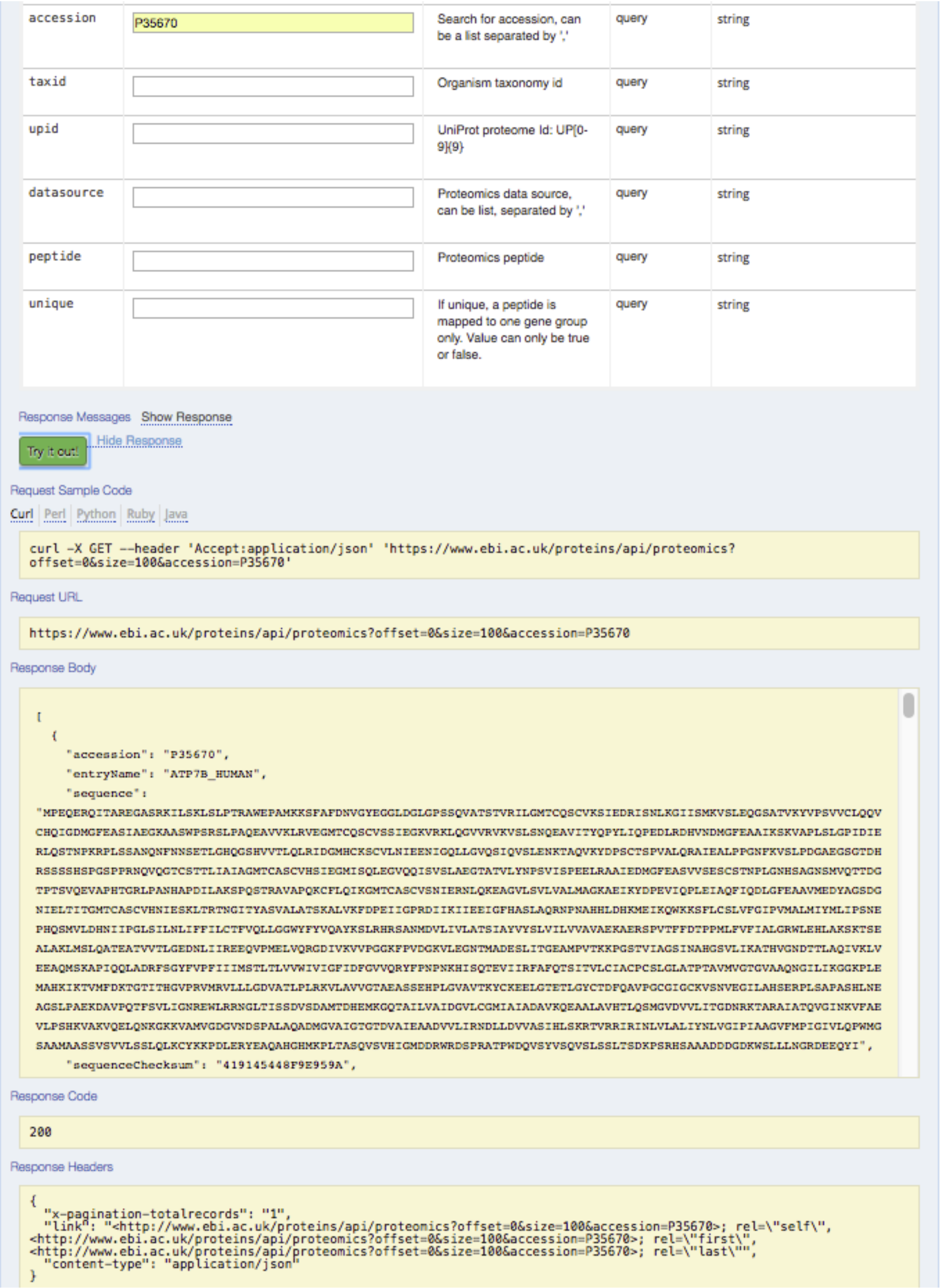

Figure 10: Results from executing the search for peptides.

```
curl -X GET --header 'Accept:application/json'
'https://www.ebi.ac.uk/proteins/api/proteomics?offset=0&size=100&accessi
on=P35670'
```

Figure 11: Curl command to execute the search for proteomics peptides dynamically generated by the Swagger UI as illustrated in Figure 10.

```
import requests, sys

requestURL =
"https://www.ebi.ac.uk/proteins/api/proteomics?offset=0&size=100&accessi
on=P35670"

r = requests.get(requestURL, headers={ "Accept" : "application/json"})

if not r.ok:
  r.raise_for_status()
  sys.exit()

responseBody = r.text
print(responseBody)
```

Figure 12: Python script equivalent to the curl command in Figure 11 for executing the search for proteins features in P35670.

Peptide position in P35670	LSS proteomics sources	Peptide sequence
1042 - 1054	MaxQB, PeptideAtlas	VLLLGDVATLPLR
1042 - 1055	MaxQB, PeptideAtlas	VLLLGDVATLPLRK
1056 - 1077	MaxQB, PeptideAtlas	VLAVVGTAEASSEHPLGVAVTK
1106 - 1137	PeptideAtlas	VSNVEGILAHSERPLSAPASHLNEAGSLPAEK
1229 - 1238	MaxQB, PeptideAtlas	AIATQVGINK
1302 - 1315	MaxQB	NDLLDVVASIHLSK
1416 - 1440	PeptideAtlas	ATPWDQVSYVSQVSLSSLTSDKPSR

Table 1: Unique peptides upstream of active site at residue 1027. Sources for the experimental evidence of the peptide are from either MaxQB or PeptideAtlas.

Step 4: Searching for ATP7B orthologues

The *Proteins* service was used to find any orthologues of human APT7B by searching by gene name. Figure 13 shows the Swagger UI for the *Proteins* service with gene name ATP7B added to the gene search field. Figure 14 illustrates the results after execution of the search in the response body, with a Curl command defined that is also available in Figure 15 with Figure 16 defining a Python script to execute this search. Figure 17 summarises the sequence identity of 83.59% between the two protein sequences when aligned by Clustal Omega. The full sequence alignment of the two protein sequences is shown in Figure 18.

```
curl -X GET --header 'Accept:application/xml'
'https://www.ebi.ac.uk/proteins/api/proteins?offset=0
&size=-1&gene=Atp7b'
```

Figure 15: Curl command to execute the search for protein orthologues dynamically generated by the Swagger UI as illustrated in Figure 16. Here due to the large amount of data returned the pagination has been 'switch-off' using the size=-1 option. Users are recommended to use the pagination option in their scripts.

```
import requests, sys

requestURL =
"https://www.ebi.ac.uk/proteins/api/proteins?offset=0
&size=-1&gene=Atp7b"

r = requests.get(requestURL, headers={ "Accept" :
"application/xml"})

if not r.ok:
  r.raise_for_status()
  sys.exit()

responseBody = r.text
print(responseBody)
```

Figure 16: Dynamically generated python script for searching for proteins from the ATP7B gene across all species. Note that in this example pagination has been switched off so all the results can be returned in a single response (size=-1). Users are recommended to use the pagination option in their scripts.

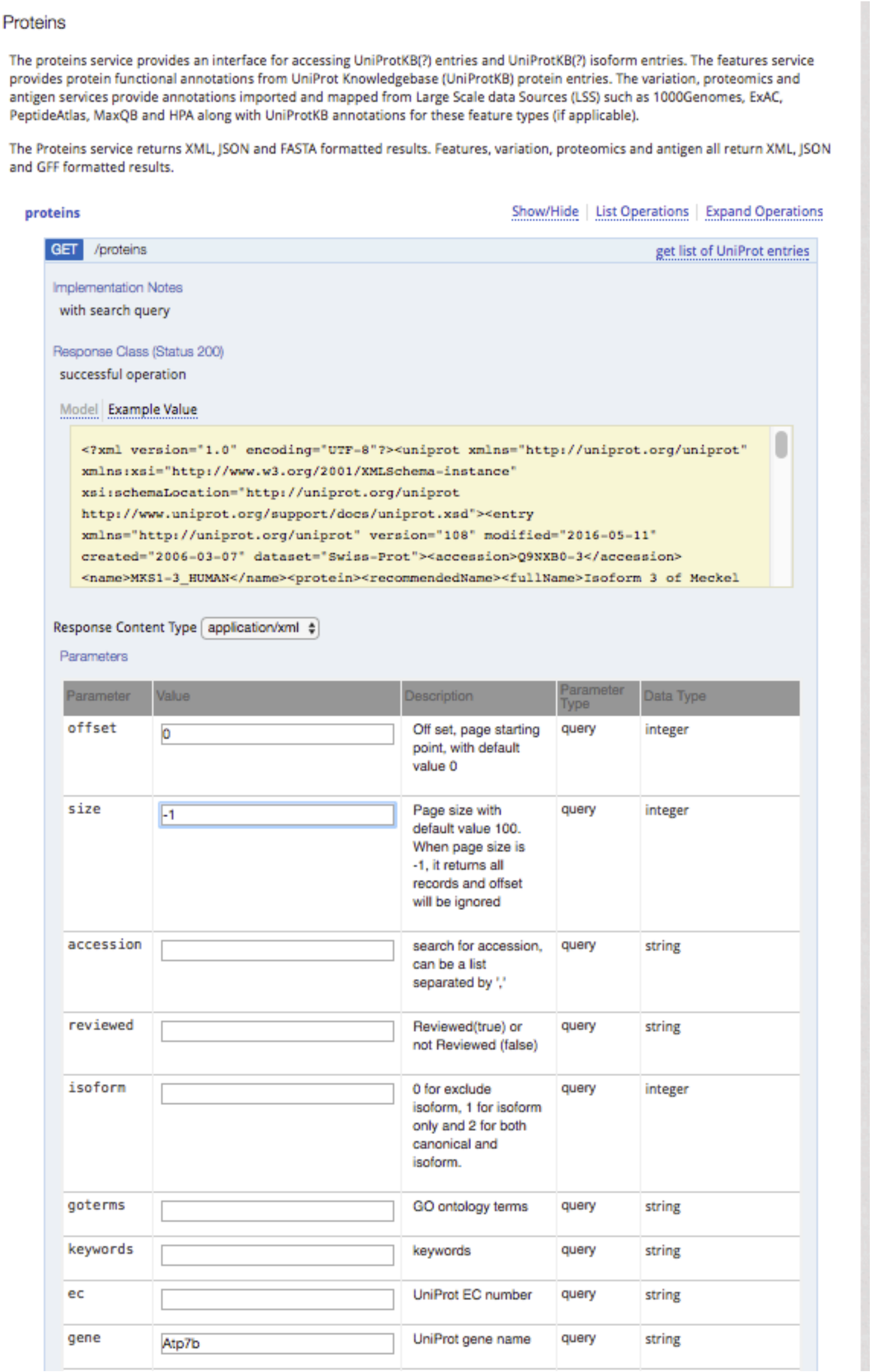

Figure 13: *Proteins* service Swagger UI with 'ATP7B' entered in to the gene search field. Note that the search is case insensitive.

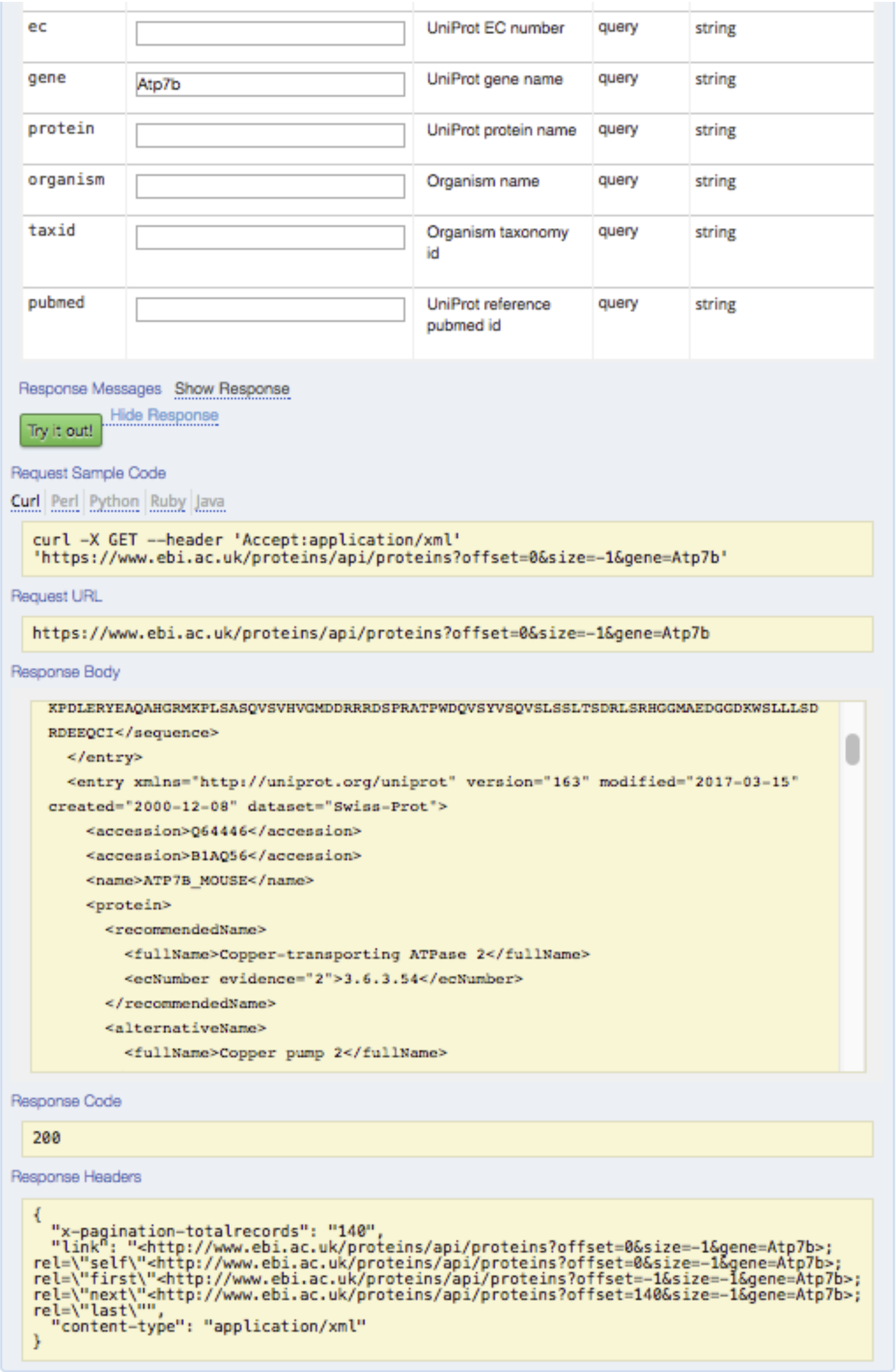

Figure 14: Results from executing the search for orthologue proteins in the *Proteins* service.

```
#
#
#  Percent Identity  Matrix - created by Clustal2.1
#
#

     1: P35670      100.00   83.59
     2: Q64446       83.59  100.00
```

Figure 17: Summary of sequence identity between human ATP7B P35670 and mouse ATP7B Q64446 proteins calculated by sequence alignment with Clustal Omega.

```
CLUSTAL O(1.2.4) multiple sequence alignment


P35670          ------------MPEQERQITAREGASRKILSKLSLPTRAWEPAMKKSFAFDNVGYEGGL    48
Q64446          MDPRKNLASVGTMPEQERQVTAKE-ASRKILSKLALPGRPWEQSMKQSFAFDNVGYEGGL    59
                            *******:**:* *********:** * ** :**:*************

P35670          DGLGPSSQVATSTVRILGMTCQSCVKSIEDRISNLKGIISMKVSLEQGSATVKYVPSVVC    108
Q64446          DSTSS-SPAATDVVNILGMTCHSCVKSIEDRISSLKGIVNIKVSLEQGSATVRYVPSVMN    118
                *. .  * .**..*.******:***********.****:.:***********:*****:

P35670          LQQVCHQIGDMGFEASIAEGKAASWPSRSLPAQEAVVKLRVEGMTCQSCVSSIEGKVRKL    168
Q64446          LQQICLQIEDMGFEASAAEGKAASWPSRSSPAQEAVVKLRVEGMTCQSCVSSIEGKIRKL    178
                ***:* ** ******* ************ **************************:***

P35670          QGVVRVKVSLSNQEAVITYQPYLIQPEDLRDHVNDMGFEAAIKSKVAPLSLGPIDIERLQ    228
Q64446          QGVVRIKVSLSNQEAVITYQPYLIQPEDLRDHICDMGFEAAIKNRTAPLRLGPIDVNKLE    238
                *****:**************************: *********.:.*** *****:::*:

P35670          STNPKRPLSSANQNFNNSETLGHQGSHVVTLQLRIDGMHCKSCVLNIEENIGQLLGVQSI    288
Q64446          STNLKKETVSPVQISNHFETLGHQGSYLATLPLRIDGMHCKSCVLNIEGNIGQLPGVQNI    298
                *** *:   *  *  *: ********::.** **************** ***** ***.*

P35670          QVSLENKTAQVKYDPSCTSPVALQRAIEALPPGNFKVSLPDGAEGSGTDHRSSSSHSPGS    348
Q64446          HVSLENKTAQIQYDPSCVTPMFLQTAIEALPPGHFKVSLPDGVEENEPQS--------GS    350
                :*********::*****.:*: ** ********:********.* .  :         **

P35670          PPRNQVQGTCSTTLIAIAGMTCASCVHSIEGMISQLEGVQQISVSLAEGTATVLYNPSVI    408
Q64446          SQRHQEQGPGRTAVLTISGITCASSVQPIEDMLSQRKGVQQTSISLAEGTGAVLYDPSIV    410
                  *:* **   *::::*:*:****.*: **.*:** :**** *:******.:***:**::

P35670          SPEELRAAIEDMGFEASVVSESCSTNPLGNHSAGNSMVQTTDGTPTSVQEVAPHTGRLPA    468
Q64446          SLDELRTAVEDMGFEVSVNSETFTINPVRNFKSGNSVPQTMGDIAGSVQKMAPDTRGLPT    470
                * :***:*:******.** **: : **: *..:***: ** ..   ***::**.*  **:

P35670          NHAPDILAKSPQSTRAVAPQKCFLQIKGMTCASCVSNIERNLQKEAGVLSVLVALMAGKA    528
Q64446          HQGPGHSSETPSSPGATASQKCFVQIKGMTCASCVSNIERSLQRHAGILSVLVALMSGKA    530
                ::.*.  :::*.*  *.* ****:****************.**:.**:********:***

P35670          EIKYDPEVIQPLEIAQFIQDLGFEAAVMEDYAGSDGNIELTITGMTCASCVHNIESKLTR    588
Q64446          EVKYDPEIIQSPRIAQLIQDLGFEASVMEDNTVSEGDIELIITGMTCASCVHNIESKLTR    590
                *:*****:**  .***:********:**** : *:*:*** *******************

P35670          TNGITYASVALATSKALVKFDPEIIGPRDIIKIIEEIGFHASLAQRNPNAHHLDHKMEIK    648
Q64446          TNGITYASVALATSKAHVKFDPEIVGPRDIIKIIEEIGFHASLAQRNPNAHHLDHKTEIK    650
                **************** *******:******************************* ***

P35670          QWKKSFLCSLVFGIPVMALMIYMLIPSNEPHQSMVLDHNIIPGLSILNLIFFILCTFVQL    708
Q64446          QWKKSFLCSLVFGIPVMGLMVYMLIPSSTPQETMVLDHNIIPGLSVLNLIFFILCTFVQF    710
                *****************.**:******. *:::************:*************:

P35670          LGGWYFYVQAYKSLRHRSANMDVLIVLATSIAYVYSLVILVVAVAEKAERSPVTFFDTPP    768
Q64446          LGGWYFYVQAYKSLRHRSANMDVLIVLATTIAYAYSLVILVVAVAEKAEKSPVTFFDTPP    770
                *****************************:***.**************:***********

P35670          MLFVFIALGRWLEHLAKSKTSEALAKLMSLQATEATVVTLGEDNLIIREEQVPMELVQRG    828
Q64446          MLFVFIALGRWLEHVAKSKTSEALAKLMSLQATEATVVTLGEDNLILREEQVPMELVQRG    830
                **************:*******************************:*************

P35670          DIVKVVPGGKFPVDGKVLEGNTMADESLITGEAMPVTKKPGSTVIAGSINAHGSVLIKAT    888
Q64446          DVIKVVPGGKFPVDGKVLEGNTMADESLITGEAMPVTKKPGSIVIAGSINAHGSVLLKAT    890
                *::*************************************** *************:***

P35670          HVGNDTTLAQIVKLVEEAQMSKAPIQQLADRFSGYFVPFIIIMSTLTLVVWIVIGFIDFG    948
Q64446          HVGNDTTLAQIVKLVEEAQMSKAPIQQLADRFSGYFVPFIIIISTLTLVVWIVIGFVDFG    950
                ******************************************:*************:***
```

```
P35670      VVQRYFPNPNKHISQTEVIIRFAFQTSITVLCIACPCSLGLATPTAVMVGTGVAAQNGIL    1008
Q64446      VVQKYFPSPSKHISQTEVIIRFAFQTSITVLCIACPCSLGLATPTAVMVGTGVAAQNGVL    1010
            ***:***.*.************************************************:*

P35670      IKGGKPLEMAHKIKTVMFDKTGTITHGVPRVMRVLLLGDVATLPLRKVLAVVGTAEASSE    1068
Q64446      IKGGKPLEMAHKIKTVMFDKTGTITHGVPRVMRFLLLADVATLPLRKVLAVVGTAEASSE    1070
            *********************************.***.**********************

P35670      HPLGVAVTKYCKEELGTETLGYCTDFQAVPGCGIGCKVSNVEGILAHSERPLSAPASHLN    1128
Q64446      HPLGVAVTKYCKEELGTETLGYSTDFQAVPGCGISCKVSNVEGILARSDLT-----AHPV    1125
            **********************.***********.***********:*:       :*

P35670      EAGSLPAEKDAVPQTFSVLIGNREWLRRNGLTISSDVSDAMTDHEMKGQTAILVAIDGVL    1188
Q64446      GVGNPPTGEGAGPQTFSVLIGNREWMRRNGLTISSDISDAMTDHEMKGQTAILVAIDGVL    1185
             .*. *: :.* *************:**********:***********************

P35670      CGMIAIADAVKQEAALAVHTLQSMGVDVVLITGDNRKTARAIATQVGINKVFAEVLPSHK    1248
Q64446      CGMIAIADAVKPEAALAIYTLKSMGVDVALITGDNRKTARAIATQVGINKVFAEVLPSHK    1245
            *********** *****::**:******.*******************************

P35670      VAKVQELQNKGKKVAMVGDGVNDSPALAQADMGVAIGTGTDVAIEAADVVLIRNDLLDVV    1308
Q64446      VAKVQELQNEGKKVAMVGDGVNDSPALAQADVGIAIGTGTDVAIEAADVVLIRNDLLDVV    1305
            *********:*********************:*:**************************

P35670      ASIHLSKRTVRRIRINLVLALIYNLVGIPIAAGVFMPIGIVLQPWMGSAAMAASSVSVVL    1368
Q64446      ASIHLSKRTVRRIRVNLVLALIYNMVGIPIAAGVFMPIGIVLQPWMGSAAMAASSVSVVL    1365
            **************:*********:***********************************

P35670      SSLQLKCYKKPDLERYEAQAHGHMKPLTASQVSVHIGMDDRWRDSPRATPWDQVSYVSQV    1428
Q64446      SSLQLKCYRKPDLERYEAQAHGRMKPLSASQVSVHIGMDDRRRDSPRATAWDQVSYVSQV    1425
            ********:*************:****:************* ******* **********

P35670      SLSSLTSDKPSRHSAAADDDGDKWSLLLNGRDEEQYI  1465
Q64446      SLSSLTSDRLSRHGGAAEDGGDKWSLLLSDRDEEQCI  1462
            ********: ***..**:*.********..***** *
```

Figure 18: Sequence alignment of human ATP7B protein P35670 and mouse ATP7B protein Q64446 by Clustal Omega. As summarised in Figure 17 the two proteins are 83.59% identical.

Step 5: Gene structure analysis using *Coordinates* service

The *Coordinates* service was used to retrieve genomic coordinates mapped to both the human APT7B protein P35670 and mouse ATP7B protein Q64446 searching by accession. Figure 19 shows the Swagger UI for the *Coordinates* service with accession P35670 added to the accession search field and the results after execution of the search in the response body, with a Curl command defined that is also available in Figure 20 with Figure 21 defining a Python script to execute this search. The equivalent coordinate search for mouse ATP7B protein Q64446 is shown in Figure 22, with the dynamically generated curl command and python script in Figures 23 and 24 respectively. Both P35670 and Q64446 genes consist of 21 exons with the active site residue in both proteins being found in the fourteenth exon. Therefore, we can be confident that other sequence features are related between the two proteins.

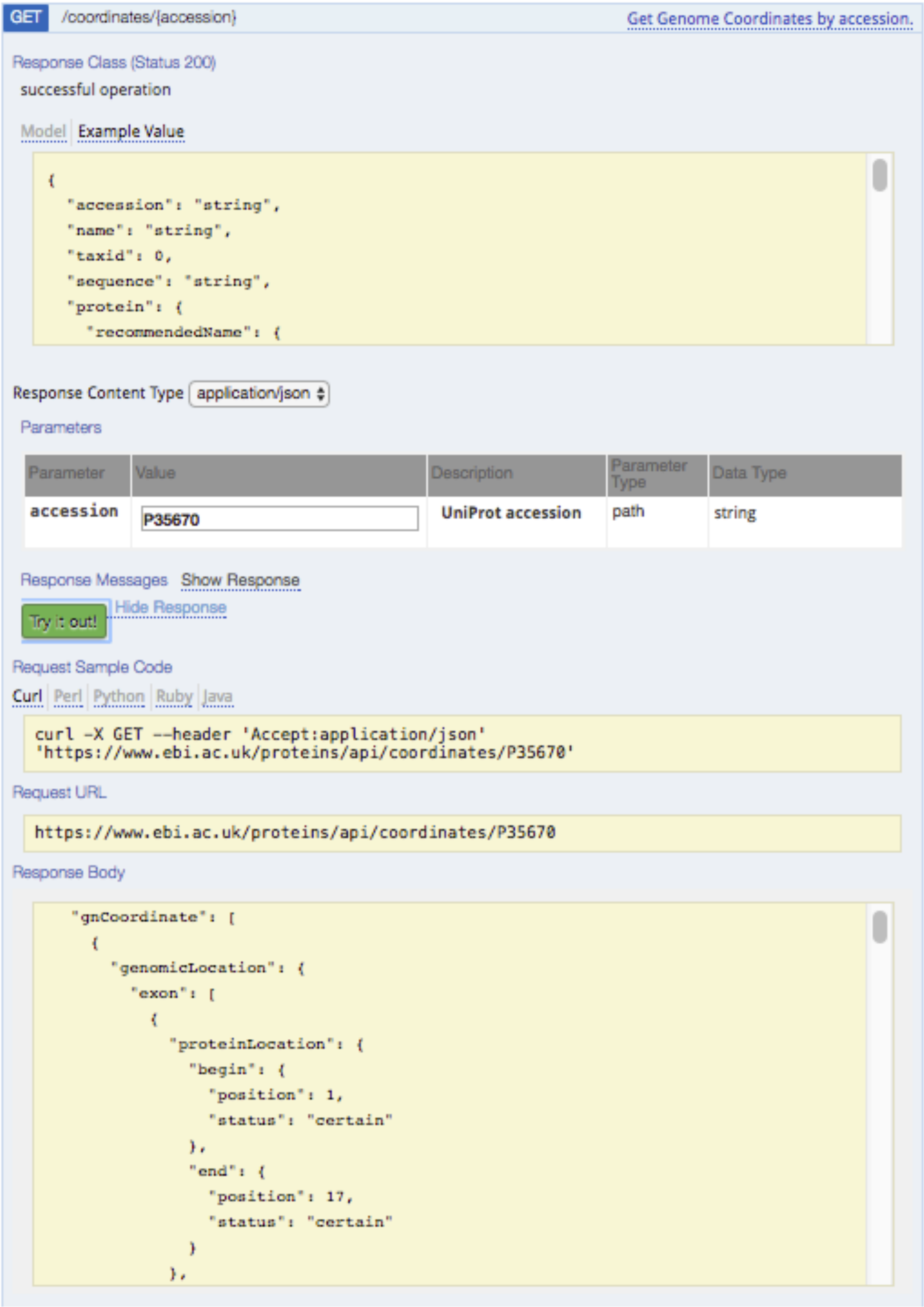

Figure 19: *Coordinates* service Swagger UI with the human protein identifier P35670 entered in to the accession search field. The results body contains the protein and matching genomic coordinates for the protein.

```
curl -X GET --header 'Accept:application/json'
'https://www.ebi.ac.uk/proteins/api/coordinates/P3567
0'
```

Figure 20: Curl command to execute the search for mapped genomic coordinates for P35670 dynamically generated by the Swagger UI as illustrated in Figure 19.

```
import requests, sys

requestURL =
"https://www.ebi.ac.uk/proteins/api/coordinates/P3567
0"

r = requests.get(requestURL, headers={ "Accept" :
"application/json"})

if not r.ok:
  r.raise_for_status()
  sys.exit()

responseBody = r.text
print(responseBody)
```

Figure 21: Dynamically generated python script for searching for the genomic coordinates for protein P35670.

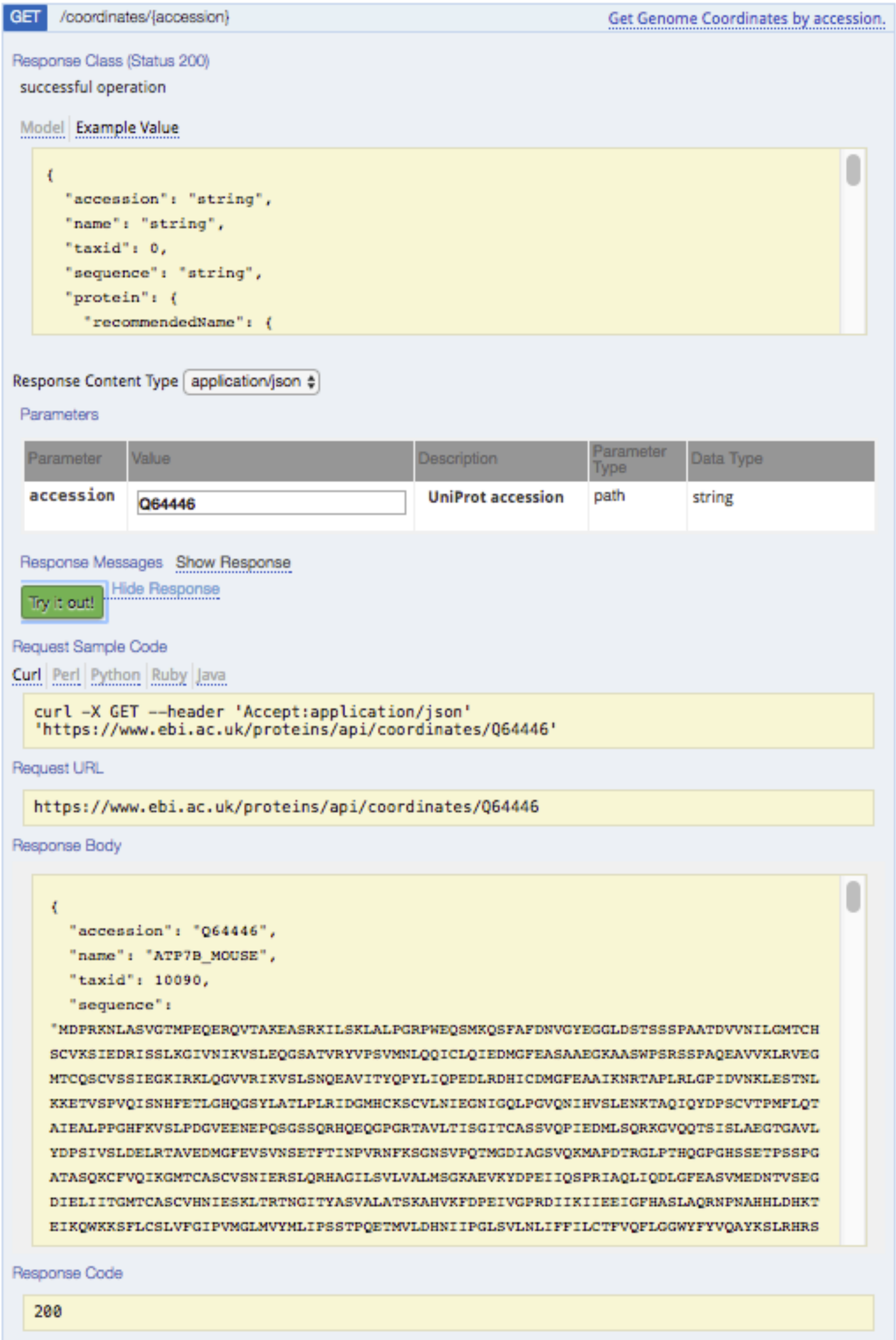

Figure 22: *Coordinates* service Swagger UI with the mouse protein identifier Q64446 entered in to the accession search field. The results body contains the protein and matching genomic coordinates for the protein.

```
curl -X GET --header 'Accept:application/json'
'https://www.ebi.ac.uk/proteins/api/coordinates/Q6444
6'
```

Figure 23: Curl command to execute the search for mapped genomic coordinates for P35670 dynamically generated by the Swagger UI as illustrated in Figure 19.

```
import requests, sys

requestURL =
"https://www.ebi.ac.uk/proteins/api/coordinates/Q6444
6"

r = requests.get(requestURL, headers={ "Accept" :
"application/json"})

if not r.ok:
  r.raise_for_status()
  sys.exit()

responseBody = r.text
print(responseBody)
```

Figure 24: Dynamically generated python script for searching for the genomic coordinates for protein Q64446.

Step 6: Mouse Q64446 variant search and comparison to P35670

The *Variation* service was used to retrieve any variants for mouse ATP7B protein Q64446 by searching with the protein accession. Figure 25 shows the Swagger UI for the *Variation* service with accession Q64446 added to the accession search field. Results after execution of the search are shown in Figure 26, where the mouse variant p.R1040K is shown as it is equivalent to the human p.R1038K Wilson disease associated variant. The curl command to execute the search is defined in Figure 27 with Figure 28 defining a Python script to execute this search.

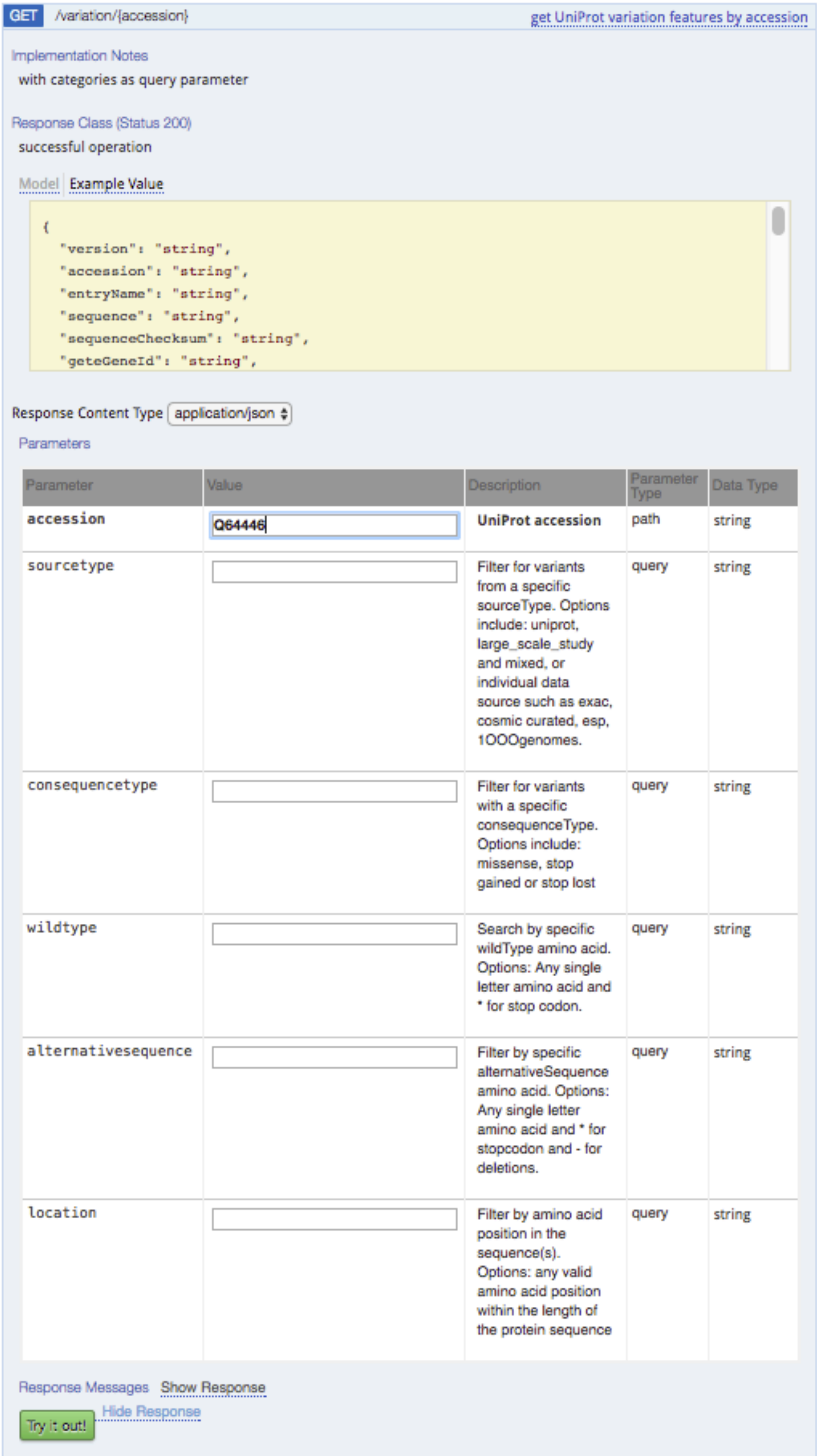

Figure 25: *Variation* service Swagger UI with the mouse protein identifier Q64446 entered in to the accession search field.

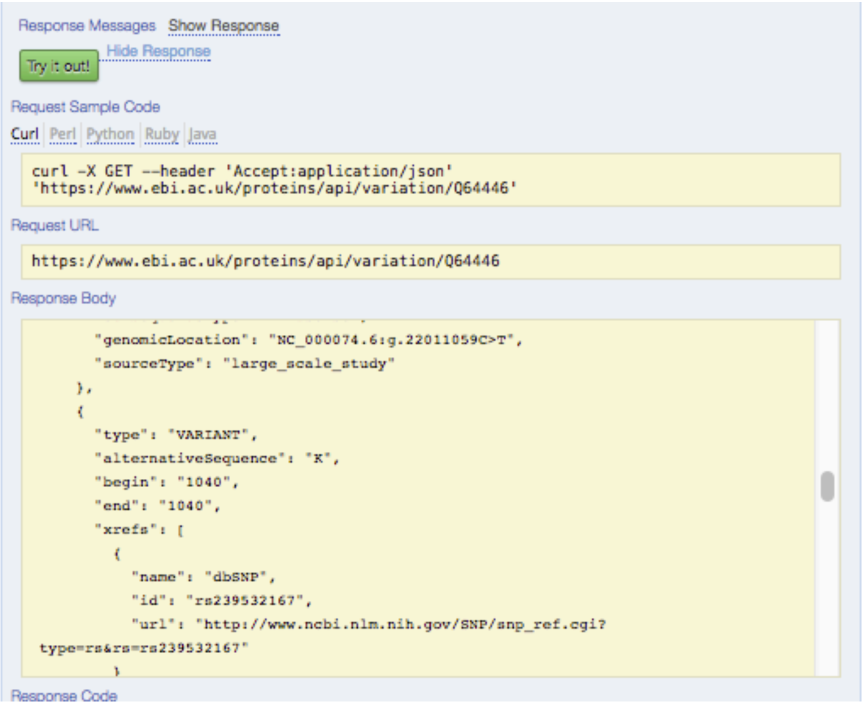

Figure 26: Results from the *Variation* search with the mouse protein identifier Q64446. In the response body is the mouse equivalent variant p.R1040K to the human p.R1038K Wilson disease associated variant.

```
curl -X GET --header 'Accept:application/json'
'https://www.ebi.ac.uk/proteins/api/variation/Q64446'
```

Figure 27: Curl command to execute the search for variant for Q64446 dynamically generated by the Swagger UI as illustrated in Figure 26.

```
import requests, sys

requestURL =
"https://www.ebi.ac.uk/proteins/api/variation/Q64446"

r = requests.get(requestURL, headers={ "Accept" :
"application/json"})

if not r.ok:
  r.raise_for_status()
  sys.exit()

responseBody = r.text
print(responseBody)
```

Figure 28: Dynamically generated python script for searching for variants for protein Q64446.

Step 7: Mouse proteomics peptide search

The *Proteomics* service was used to retrieve any peptides for mouse ATP7B protein Q64446 by searching with the protein accession. Figure 29 shows the Swagger UI for the *Proteomics* service with accession Q64446 added to the accession search field. Results after execution of the search are shown in Figure 30, where the mouse protein has an equivalent unique peptide between residues 1044-1057 to the unique peptide 1042-1054 in the human ATP7B protein P35670. Where a peptide is defined as unique if it not found in another protein, not including isoforms, in the organisms' proteome. Unique peptides from different species can be the same. The curl command to execute the search is defined in Figure 31 with Figure 32 defining a Python script to execute this search.

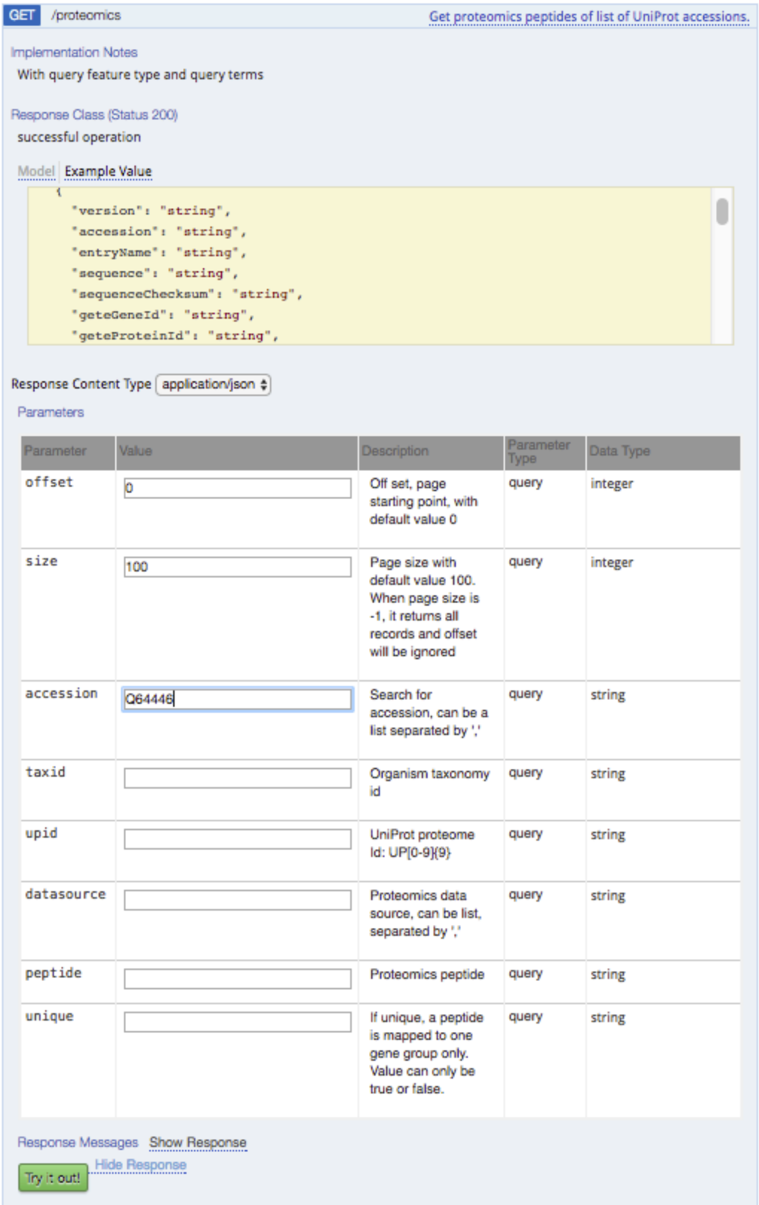

Figure 29: *Proteomics* service Swagger UI with the mouse protein identifier Q64446 entered in to the accession search field.

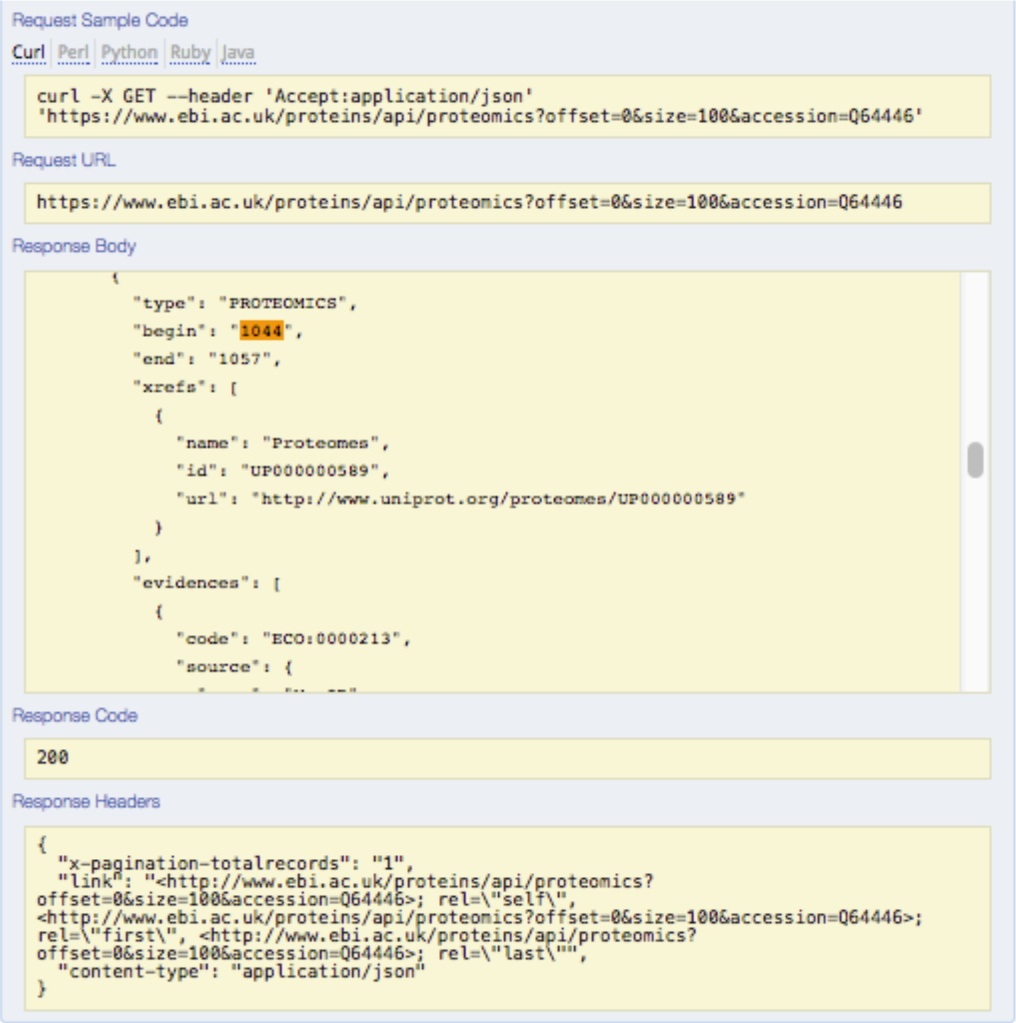

Figure 30: Results from the *Proteomics* search with the mouse protein identifier Q64446. In the response body is the mouse unique peptide starting at residue 1044.

```
curl -X GET --header 'Accept:application/json'
'https://www.ebi.ac.uk/proteins/api/proteomics?offset
=0&size=100&accession=Q64446'
```

Figure 31: Curl command to execute the search for peptides for Q64446 dynamically generated by the Swagger UI as illustrated in Figure 30.

```
import requests, sys

requestURL =
"https://www.ebi.ac.uk/proteins/api/proteomics?offset
=0&size=100&accession=Q64446"

r = requests.get(requestURL, headers={ "Accept" :
"application/json"})

if not r.ok:
  r.raise_for_status()
  sys.exit()

responseBody = r.text
print(responseBody)
```

Figure 32: Dynamically generated python script for searching for unique peptides for protein Q64446.
